# Development of a Rapid and Sensitive Visual Pesticide Detection Card Using Crosslinked and Surface-Decorated Electrospun Nanofiber Mat

**DOI:** 10.3390/foods14152628

**Published:** 2025-07-26

**Authors:** Yunshan Wei, Huange Zhou, Jingxuan Kang, Yongmei Wu, Kun Feng

**Affiliations:** 1 College of Food Science and Engineering, Henan University of Technology, Zhengzhou 450001, China; 2 College of Food and Bioengineering, Zhengzhou University of Light Industry, Zhengzhou 450001, China; 3 Key Laboratory of Cold Chain Food Processing and Safety Control, Zhengzhou University of Light Industry, Ministry of Education, Zhengzhou 450001, China

**Keywords:** nanofiber mat, surface crosslinking, pesticide detection, rapid and sensitive

## Abstract

Increased consumer awareness on food safety has spurred the development of detection techniques for pesticide residues. In this study, a rapid detection card on the basis of enzyme action was developed for the visual detection of pesticides, in which the thermally crosslinked and surface-decorated polyvinyl alcohol/citric acid nanofiber mat (PCNM) was employed as a novel immobilization matrix for acetylcholinesterase (AChE). The PCNM, crosslinked at 130 °C for 50 min, exhibited appropriate microstructure and water stability, making it suitable for AChE immobilization. The activation of carboxyl groups by surface decoration resulted in a 2.5-fold increase in enzyme loading capacity. Through parameter optimization, the detection limits for phoxim and methomyl were determined to be 0.007 mg/L and 0.10 mg/L, respectively. The detection card exhibited superior sensitivity and a reduced detection time (11 min) when compared to a commercially available pesticide detection card. Furthermore, the detection results of pesticide residues in fruit and vegetable samples confirmed its feasibility and superiority over commercial alternatives, suggesting its great potential for practical application in the on-site detection of pesticide residues.

## 1. Introduction

Pesticides have played a critical role in ensuring both crop yield and quality. However, the excessive and uncontrolled use of pesticides poses a significant threat to sustainable food production and food safety [1]. Consequently, on-site monitoring of pesticide residues in agricultural products is essential for ensuring global food safety and environmental protection. Traditional techniques such as high-performance liquid chromatography and gas chromatography demonstrate commendable sensitivity and accuracy in pesticide detection [2]. However, factors including complex equipment operation, time-intensive detection processes, and high costs restrict their suitability for on-site or field detection of pesticide residues in food [3]. In addition, these methods are inaccessible to grassroots consumers, including farmers, who seek to identify pesticide residues in food through simple and qualitative on-site assays. In this regard, rapid pesticide detection technologies offer quick, easy-to-operate, and cost-effective alternatives that can serve as supplements to conventional methods. In recent years, research into rapid pesticide detection employing principles such as colorimetric, electrochemical, and optical sensing strategies has garnered significant attention [4]. Notably, colorimetric sensing represents non-label visual detection methods that enable the real-time screening of pesticide residues with the naked eye, typically without requiring trained technicians or professional equipment. In recent years, enzyme-mediated colorimetric assays have attracted particular attention. Among the enzymes employed in paper-based devices, acetylcholinesterase (AChE) is the most widely adopted compared to the other typically used enzyme butyrylcholinesterase (BChE) [5]. The visual detection method operates through pesticide-induced inhibition of AChE, which suppresses the enzyme-catalyzed chromogenic reaction of substrates (such as indoxacarb) [6]. This simple color change is employed to qualitatively identify pesticide residues and has been widely utilized in commercial rapid detection cards. Most studies on visual detection focus on the activity of acetylcholinesterase (AChE) enzymes. For these rapid detection cards, the immobilization of enzymes is closely linked to the carrier structure, which is a crucial factor affecting their detection performance. Currently, the primary materials used for pesticide detection cards include qualitative filter paper, absorbent paper, and cast films. However, the detection time and sensitivity of these materials increasingly fail to meet the stringent market demands for food pesticide detection. In this context, nanomaterials have emerged as ideal carriers for enzyme immobilization due to their high specific surface area and porosity, presenting significant potential for application in the research of rapid detection cards [7].

Recently, electrospinning has gained prominence as a versatile route to produce nanofibers [8]. Electrospun nanofiber mats exhibit superior properties as biosensor matrices due to their porous architecture and high surface area [9]. These characteristics confer a high loading capacity for biocompounds, enhance the diffusion of target analytes to the active sites, and increase the number of interaction sites, thereby leading to improved biosensor sensitivity [10]. It has been highlighted that the thermal stability of enzymes immobilized on electrospun nanofilms exceeds that of enzymes on coated films and their free form [11]. Although the potential application of electrospun nanofiber mats for enzyme immobilization has been demonstrated [12], their use as matrices for constructing rapid detection cards remains limited. Our team previously reported, for the first time, the feasibility of utilizing electrospun nanofiber mats for the construction of rapid detection cards [13,14]. However, several issues persist concerning the application of these carriers: (1) carriers made from hydrophilic materials, such as polyvinyl alcohol (PVA), tend to swell and deform upon exposure to water during the preparation and detection processes; and (2) the construction of hydrophobic carriers from hydrophobic polymers (e.g., PCL) can mitigate these issues; however, it necessitates the use of organic solvents during fiber mat preparation and involves harsh modification treatments of fiber mats. Consequently, reducing the hydrophilicity of carriers and enhancing enzyme adsorption through environmentally friendly methods represents a significant avenue for addressing these challenges. Citric acid (CA), a tricarboxylic organic acid, readily engages in covalent or ionic interactions with hydroxyl and amine groups present in biopolymers [15]. Our ongoing research indicates that CA crosslinking effectively reduces the hydrophilic swelling of PVA fiber mats. Furthermore, the incorporation of CA enriches the surface of the fiber mat with additional carboxyl groups, thereby facilitating the covalent adsorption of enzymes [16]. However, to date, the feasibility of this strategy remains unknown and warrants further investigation.

Herein, in this study, a crosslinked and decorated nanofiber mat was developed for the immobilization of acetylcholinesterase (AChE), resulting in the creation of an enzyme card (EC) for detection purposes. To enhance the water stability of the fiber mat, thermal crosslinking of the PVA/CA nanofiber mat (PCNM) was performed. Subsequently, the carboxyl groups of the crosslinked fiber mat were activated through surface decoration with 1-ethyl-(3-dimethylaminopropyl) carbodiimide hydrochloride (EDC)/N-hydroxysuccinimide (NHS) (EDC/NHS, EN). The properties of the nanofiber mat for pesticide detection were characterized and evaluated. Furthermore, key factors such as the enzyme concentration and the inhibition and color development times were optimized to enhance the detection performance of the card. The corresponding pesticide detection limits were subsequently determined. Finally, the efficacy of this method for real food samples was evaluated in comparison with a commercial card. This study offers a green route to fabricate nanoscale carriers enabling rapid, sensitive pesticide assays and is anticipated to spur broader adoption of electrospinning within the food and agriculture sectors.

## 2. Materials and Methods

### 2.1. Materials

Phoxim and methomyl were purchased from a local pesticide shop, and PVA, acetylcholinesterase (AChE) (200 U/g), citric acid (CA), 1-ethyl-(3-dimethylaminopropyl) carbodiimide hydrochloride (EDC), and N-hydroxysuccinimide (NHS) were purchased from Shanghai Maclean’s Biochemical Technology Co., Ltd. (Shanghai, China). Indolyl acetate (IA) was purchased from Shanghai Yuanye Biotechnology Co., Ltd. (Shanghai, China). Qualitative filter paper was purchased from Hunan Bickman Holdings Co., Ltd. (Changsha, China). Dipotassium phosphate and potassium dihydrogen phosphate were purchased from Tianjin Kemio and Tianjin Zhiyuan Chemical Reagent Co., Ltd. (Tianjin, China).

### 2.2. Preparation and Thermal Crosslinking of PCNM

PVA solutions with concentrations of 5%, 7.5%, 10%, and 12.5% were prepared by dissolving PVA into deionized water while continuously stirring at 90 °C for 4 h. Subsequently, CA was incorporated into the PVA solutions at a concentration of 20% (wt./PVA weight), with constant stirring maintained for an additional 2 h. The conductivity and viscosity of different solutions were measured using a conductivity meter (DDS-307A, Inesa Scientific Instrument Co., Ltd., Shanghai, China) and a viscometer (LV-SSR, Shanghai Fangrui Instrument Co., Ltd., Shanghai, China), respectively. Electrospinning was conducted at a voltage range of 13–17 kV, a flow rate of 0.25–0.65 mL/h, and a distance of 11–15 cm. PVA nanofiber mats (PNMs) and PVA/CA nanofiber mats (PCNMs) were collected over 10 h at 25 ± 2 °C with a relative humidity of 50 ± 2%.

To initiate crosslinking, the PCNM (approximately 0.15 mm) was heated in an oven. The thermal crosslinking was performed either within a temperature range of 110 °C to 150 °C for 50 min or at 130 °C for 10-70 min, respectively. Subsequently, the crosslinked PCNM (C-PCNM) was stored in a dryer prior to characterization and its application in the construction of the detection card.

### 2.3. Construction of Detection Card Using Surface-Decorated C-PCNM

**Scheme 1:**
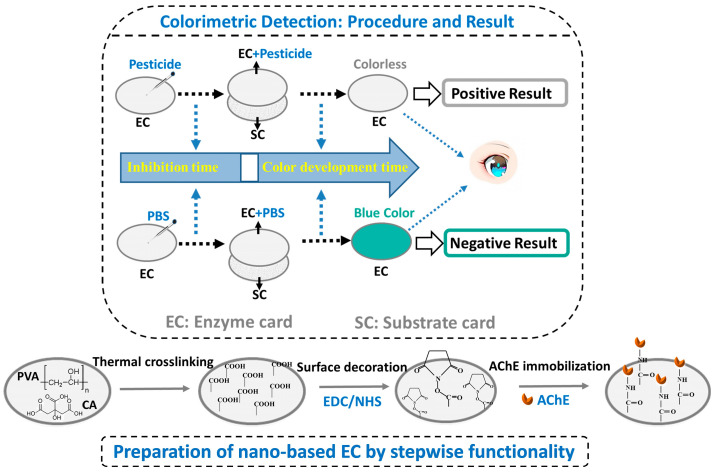
Procedure of the colorimetric detection card and the fabrication of the nano-based enzyme card by stepwise functionality.

In this work, the detection platform comprises an enzyme card (EC) and a substrate card (SC). The stepwise functionality of the crosslinked PCNM and its application in constructing the EC are illustrated in Scheme 1. To enhance the immobilization sites for AChE, surface decoration of the crosslinked nanofiber mat was conducted using EDC/NHS (EN). Briefly, the fiber mat with a diameter of 1 cm was immersed in a 1 M EN PBS solution at 4 °C for a duration of 1 to 4 h. After rinsing with PBS, the decorated nanofiber mat (E-PCNM) was incubated in a 1 mg/mL AChE PBS solution (pH 7.4) at 4 °C for 1.5 h to form the EC. The effect of the EN treatment on AChE immobilization was evaluated using a BCA protein assay kit, and the corresponding detection performance of the EC was assessed using qualitative filter paper (1 cm) immersed in a 3 mg/mL indoxyl acetate (IA) ethanol solution as the SC.

Additionally, the influences of the AChE concentration (0.5, 0.7, 1.0 mg/mL) and absorption time (0.5 to 2 h) on the detection efficacy of pesticide were investigated. Throughout the experiment, PBS served as the control, while a concentration of 0.01 mg/L phoxim was used as the positive sample.

### 2.4. Stability of AChE After Immobilization

The stability of the enzyme during the preparation procedure is a critical factor influencing the efficiency of the detection card. Therefore, the integrity and activity of AChE following immobilization were examined. Initially, circular dichroism (CD) measurements were conducted with a quartz cell (1 cm path length) to examine the secondary structure of both free AChE and AChE released from the EN-decorated PCNM (E-PCNM). Circular dichroism (CD) spectra were recorded over the wavelength range of 190 to 260 nm, utilizing a bandwidth of 1 nm, a step resolution of 1 nm, and an integration time of 1 s. Data processing was conducted using CDNN 2.1 software. Additionally, the activity of various AChE samples was assessed using an AChE activity assay kit (Jiangsu Addison Biotechnology Co., Ltd., Yancheng, China).

### 2.5. Characterization of Electrospun Nanofiber Mat

#### 2.5.1. Scanning Electron Microscopy (SEM)

The morphologies of PVA nanofibers, PVA/CA nanofibers, crosslinked PC nanofiber mats, and enzyme-loaded nanofiber mats were examined using SEM (Hitachi, Tokyo, Japan). Additionally, the immobilization of AChE on the PCNM and the E-PCNM was compared. The electrospun nanofiber mat (approximately 3 × 5 mm) was Au-sputtered under vacuum and observed at an accelerating voltage of 15 kV. The fiber diameter was analyzed with Image-J 2.9.0 (Bethesda, MD, USA) from approximately 50 fibers in the micrographs.

#### 2.5.2. Attenuated Total Reflectance–Fourier Transform Infrared Spectroscopy (ATR-FTIR)

The interactions among the components were analyzed using a Nicolet iS50 FTIR spectrometer (Thermo Fisher Scientific, Waltham, MA, USA) in attenuated total reflection mode. All measurements were performed over a wavenumber range of 4000 to 500 cm^−1^, with a resolution of 4 cm^−1^.

#### 2.5.3. Thermogravimetric Analysis (TGA)

The thermal properties of the electrospun nanofiber mat and other components were evaluated via a thermal gravimetric analyzer (Perkin Elmer, Waltham, MA, USA). Approximately 2 mg of samples was sealed in aluminum pans and heated from 35 to 800 °C at 10 °C/min under a nitrogen atmosphere. The Pyris™ 11.1 software platform was used for data processing.

#### 2.5.4. Mechanical Analysis 

The mechanical behavior of the nanofiber mat was evaluated via the tensile strength and elongation at break tests using a computerized electronic universal testing machine (WDW-5, Jinan Zhongzheng Testing Machine Manufacturing Co., Ltd., Jinan, China). Prior to testing, the electrospun nanofiber mat was conditioned in a controlled laboratory environment for 24 h and then cut into rectangular samples measuring 50 mm × 10 mm. The samples were subsequently positioned between the tensile grips with an initial distance of 30 mm, and the tests were processed at a stretching rate of 50 mm/min.

### 2.6. Water Resistance Performance

#### 2.6.1. Swelling Property

The swelling behavior of the nanofiber mat was assessed following Yu’s protocol [17]. First, 3 cm × 3 cm squares of samples were dried to constant mass (*M*_0_). After 24 h of immersion in water, the surface moisture of the samples was removed, and the weight of the nanofiber mat was recorded as *M*_1_. Subsequently, the wet nanofiber mat was dried in an oven until it reached a constant weight (*M*_2_). The swelling ratio and swelling loss were then calculated using the following formulas.

$$Swelling\;rate\left(\%\right)=\frac{M_{1}-M_{0}}{M_{0}}\times 100\%$$

$$Swelling\;loss\left(\%\right)=\frac{M_{0}-M_{2}}{M_{0}}\times 100\%$$

#### 2.6.2. Contact Angle

The contact angles were measured with a DSA25 goniometer (KRüSS, Hamburg, Germany). The samples were adhered to glass slides, and a 5 μL water droplet was subsequently deposited on the surface. The contact angles were recorded and averaged from three distinct positions on the fiber mats.

### 2.7. Detection Conditions of Pesticides

Based on the principles underlying this visual detection, the inhibition and color development times are pivotal variables governing the outcomes. The inhibition time refers to the duration of the reaction between the samples and the enzyme, while the color development time denotes the period required for a color change following the interaction of the EC and SC, which occurs due to the reaction of AChE and IA. In this study, the inhibition time was optimized within the range of 0 to 12 min, with the color development time fixed at 15 min. Conversely, an inhibition time of 4 min was established to determine the appropriate color development time, which varied from 3 to 15 min. Phosphate-buffered saline (PBS) at pH 7.4 and a concentration of 0.01 mg/L phoxim were utilized as the control and positive sample, respectively. The color signal generated by the reaction of AChE with the indoxyl acetate substrate can be determined by the naked eye for a qualitative analysis. Additionally, the image was captured using a digital camera, and the color changes were determined by using the automatic colorimeter (SC-80C, Beijing Kangguang Instrument Co., Ltd., Beijing, China). The values of *L* *, *a* *, and *b* * were recorded. Before measurement, the colorimeter needs to be calibrated with a white board. The total color difference (ΔE) was calculated based on the following formula:

$$∆E=\sqrt{{\left(L*-L_{0}*\right)}^{2}+{\left(a*-a_{0}*\right)}^{2}+{\left(b*-b_{0}*\right)}^{2}}$$

where *L*_0_*, *a*_0_*, and *b*_0_* are the initial color parameters; L*, a*, and b* are the color parameters at different times.

### 2.8. Sensitivity of the Nano-Based Card

To assess the performance of the detection card, the limits of detection for two representative pesticides, namely, organophosphorus (phoxim) and carbamate (methomyl), were determined. Each pesticide was serially diluted using PBS, and 50 μL aliquots were applied to the EC for analysis, using PBS alone as the control. All assays were conducted in accordance with the conditions optimized in the above section.

### 2.9. Storage Stability of Detection Card

A batch of detection cards was stored at both 4 °C and 25 °C for a duration of 90 days to evaluate their storage stability. A 0.1 M PBS solution was used as the control, while 0.01 mg/L phoxim and 0.15 mg/L methomyl served as positive controls to assess the sensitivity at specified intervals.

### 2.10. Detection in Real Food

The real test was performed according to Sun’s method with some modification [18]. In brief, the methomyl solutions were prepared at concentrations of 0, 0.1, 0.15, and 0.2 mg/L by diluting with PBS. Based on the weight of pesticide-free Chinese cabbage and oranges, corresponding volumes of methomyl solution were sprayed onto the surface of the samples at a ratio of 1 mL/g. After being set aside at room temperature for 24 h, a portion of the samples was cut into small pieces, and 5 g of each sample was immersed in 10 mL of PBS and then shaken vigorously. After standing for 5 min, the supernatant was analyzed using both a nano-based detection card and a commercial rapid detection card.

### 2.11. Statistical Analysis

Data analysis was performed with SPSS 16.0. The differences between groups were evaluated by one-way ANOVA. Differences were considered significant if *p* < 0.05.

## 3. Results and Discussion

### 3.1. The Effect of Thermal Crosslinking on the Properties of PCNM

Nanofibers with optimal morphology are essential for the successful immobilization of enzymes to create the EC. PVA is a low-cost, eco-friendly polymer characterized by good spinnability; however, its high hydrophilicity nature constrains its utility as an immobilization matrix for the production of detection cards. In this context, our previous research demonstrated the feasibility of using hydrophobic polylactic acid (PLA) nanofiber mats as a matrix for detection cards [14]. Nonetheless, the preparation of these fiber mats involved toxic organic agents as electrospinning solvents. Therefore, in this study, thermally induced crosslinking with CA was used as a green approach to enhance the water resistance of electrospun PVA nanofiber mats. During the electrospinning process, the properties of the spinning solution, such as the viscosity, conductivity, and surface tension, determine its electrospinnability, which directly influences the microstructure of the resulting fibers [19]. Consequently, the impacts of the PVA concentration on the solution properties and the microstructure of the resulting fibers are presented in Table S1 and Figure S1, respectively. A 10% PVA concentration fabricated under 13 kV, 0.25 mL/h, and 11 cm was identified as the optimal choice, resulting in uniform and staggered PC fibers as illustrated in Figure S1. However, it was observed that the PCNM still swells and breaks in PBS. This issue can be mitigated through a thermal process that facilitates the esterification reaction between PVA and CA at elevated temperatures. The multi-carboxylic structure of CA promotes esterification reactions between its carboxyl groups and the hydroxyl groups of PVA [20]. In this regard, the effects of the crosslinking temperature and heating time on the properties of the crosslinked PCNM after immersion in PBS were investigated.

#### 3.1.1. Effect of Crosslinking Temperature on the Properties of PC Nanofiber Mat

**Figure 1:**
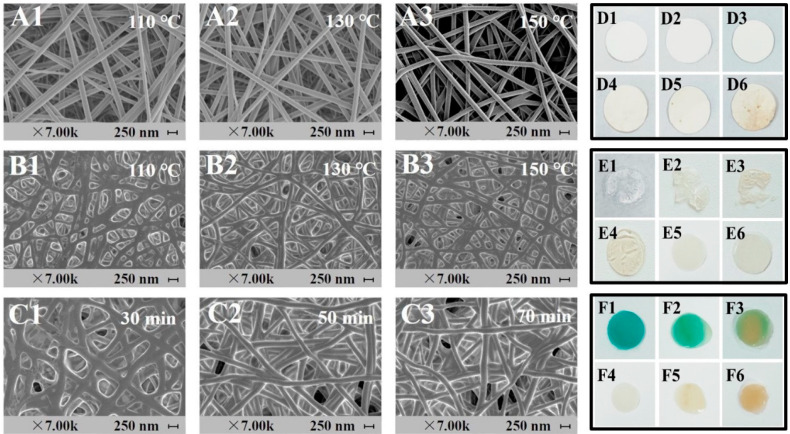
Morphology of fibers treated at different crosslinking temperatures. (**A1**–**A3**): SEM images of C-PCNM crosslinked at 110 °C, 130 °C, and 150 °C for 50 min; (**B1**–**B3**): SEM images of C-PCNM crosslinked at 110 °C, 130 °C, and 150 °C for 50 min after PBS immersion; (**C1**–**C3**): SEM images of C-PCNM crosslinked at 130 °C for 30, 50, and 70 min after PBS immersion; (**D1**–**D6**): Appearance characteristics of C-PCNM crosslinked at 100 °C, 110 °C, 120 °C, 130 °C, 140 °C, and 150 °C for 50 min; Appearance characteristics of PCNM (**E1**) and C-PCNM crosslinked at 130 °C for 10, 20, 30, 40, and 50 min after PBS immersion (**E2**–**E6**); Images of EC prepared with C-PCNM treated at 130 °C, 140 °C, and 150 °C for 50 min before (**F4**–**F6**) and after color development (**F1**–**F3**).

The fiber morphology was not significantly affected by temperature treatments ranging from 110 to 150 °C for 50 min (Figure 1(A1–A3)). Notably, the microstructural integrity of the PCNM after thermal crosslinking was substantially enhanced when immersed in PBS (Figure 1(B1–B3)), which is crucial for its application as an enzyme immobilization matrix. As shown in Figure 1(B1), the PCNM heated at 110 °C for 50 min became swollen and sticky, along with some cracks appearing on the fiber surface and an uneven distribution morphology of fibers after being soaked in PBS solution. This phenomenon may be due to the incomplete esterification of PVA with CA under these conditions [21]. In contrast, when the crosslinking temperature reached 130 °C or higher, a clear and orderly fiber morphology was observed (Figure 1(B2,B3)). Furthermore, the effect of the crosslinking temperature on the appearance characteristics of the crosslinked PCNM was examined. As depicted in Figure 1(D1-D4), the color of the nanofiber mat treated at 100 °C, 110 °C, 120 °C, and 130 °C for 50 min did not change significantly. However, the water stability of the fiber mats treated at 100 °C, 110 °C, and 120 °C was poor, leading to damage or shrinkage upon contact with PBS. In comparison, the nanofiber mat heated at 130 °C maintained good structural morphology after contact with PBS, with no color interference caused by thermal crosslinking. When the temperature exceeded 130 °C, the nanofiber mats gradually turned yellow (Figure 1(D5,D6)), and surface scorching was found at 150 °C. This change in color also negatively impacted the appearance of the detection card and the recognition of the detection result (Figure 1(F3,F6)).

#### 3.1.2. Effect of Crosslinking Time on the Properties of PCNM

The influence of the crosslinking time on the microstructure and water stability of the PCNM was investigated. As illustrated in Figure 1(E1), the PCNM disintegrated after immersion in PBS due to the strong hydrophilic nature of PVA. Similarly, PCNM treated at 130 °C for 10–30 min either sustained damage or exhibited a gel-like consistency after 12 h of immersion in PBS solution (Figure 1(E2–E4)), indicating insufficient esterification under these conditions. In contrast, when the crosslinking time was extended to 50 or 70 min, the morphology of the crosslinked fibers improved (Figure 1(C2,C3)). The nanofiber mat crosslinked at 130 °C for 40 min remained flat but rapidly shrank upon contact with PBS (Figure 1(E5)). In comparison, the surface of the nanofiber mat treated at 130 °C for 50 min was smooth and did not exhibit shrinkage, suggesting that the PCNM crosslinked for this duration had excellent water stability. These phenomena were in line with the results of the contact angle and swelling tests, which demonstrated a significant improvement in the crosslinked PCNM, as well as a reduction in absorption loss when compared to both the PNM and uncrosslinked PCNM (Table S2).

### 3.2. Property of the Enzyme Card (EC) Prepared by Surface Decoration

As part of the detection system, the construction and properties of the enzyme card (EC) require more concern due to the susceptibility of enzymes to degradation and loss of bioactivity during the preparation process. In this sense, the EC was designed by immobilizing enzymes on crosslinked PCNM, with surface decoration performed using EN to enhance enzyme immobilization. Herein, the effect of surface decoration of C-PCNM on the immobilization of AChE and its corresponding detection efficacy for pesticides was investigated.

#### 3.2.1. Effect of Surface Decoration on AChE Immobilization

**Figure 2:**
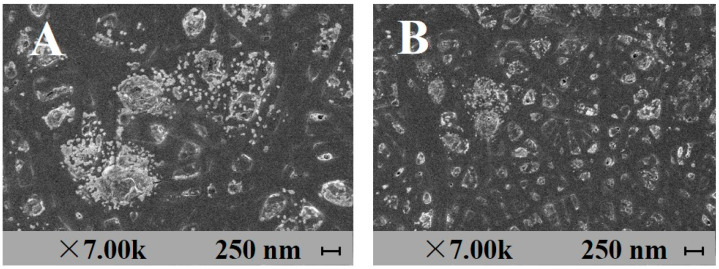
SEM images of AChE distribution in the nanofiber mat: (**A**): AChE-loaded E-PCNM; (**B**): AChE-loaded C-PCNM.

The surface activation of the C-PCNM by EN enhanced the immobilization of AChE by 2.5 times (0.064 u/each film) compared to that of the undecorated C-PCNM. This finding aligns with the observed morphologies of the enzyme-loaded nanofiber mats. As depicted in Figure 2, a substantial quantity of enzymes was immobilized on the surface of the undecorated nanofiber mat, which can be attributed to its higher specific surface area. A previous study has also demonstrated that the unique structure of nanofiber mats facilitates the loading of bioactive ingredients [22]. However, in comparison, a greater amount of enzyme was observed on the surface and within the interstitial spaces of the decorated fiber mat. This phenomenon can be explained by the activation of carboxyl groups in the C-PCNM through EN treatment, which enhances the interaction between the enzyme and the nanofiber mat. A similar result was reported in another study where lipase was immobilized via EDC/NHS-mediated amide formation between the amino groups of the lipase and the carboxyl groups of the esterified loofah sponge [23].

**Figure 3:**
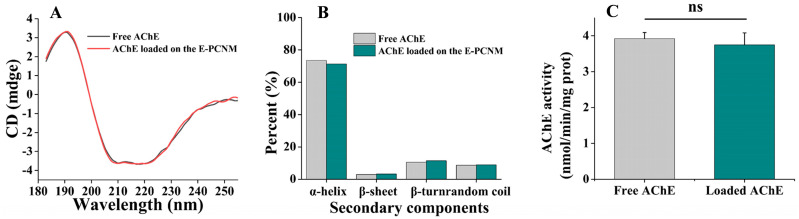
Structure and activity of free and immobilized enzymes. (**A**) CD spectra; (**B**) Secondary components; (**C**) AChE activity. ns = no significance.

Another vital aspect to consider is the activity of the enzymes immobilized on the carriers. It is well known that protein structure is a key factor influencing bioactivity [24]. In this case, the secondary structure of both free and immobilized enzymes was first measured using the far-UV circular dichroism (CD) method. As illustrated in Figure 3A, both native and immobilized AChE exhibited two negative CD absorption bands at approximately 210 and 222 nm, attributed to π-π * and n-π * transitions in amide groups, which typifies the α-helix structure [25]. The fractions of different types of secondary structures were analyzed and are shown in Figure 3A. A previous study reported that elevated α-helix content is associated with a more compact AChE structure, which may impede substrate access to the active site and consequently lead to a reduction in AChE catalytic activity [26]. However, the α-helix content for the immobilized AChE was very close to that of neat AChE (17.2%), suggesting that immobilization had minimal influence on the secondary structure of AChE. This finding is aligned with the results of the bioactivity test presented in Figure 3C, which showed no significant difference in bioactivity among AChEs immobilized on different nanofiber mats. Notably, 95.7% of bioactivity was maintained for the immobilized AChE compared to the neat AChE. Thus, it can be concluded that surface decoration has a negligible effect on the structure of AChE, allowing the immobilized AChE to retain most of its bioactivity, thereby illustrating its promising potential for use in detection cards.

#### 3.2.2. Effect of Decoration Time and Absorption Time on the Detection Efficacy of Pesticides

**Figure 4:**
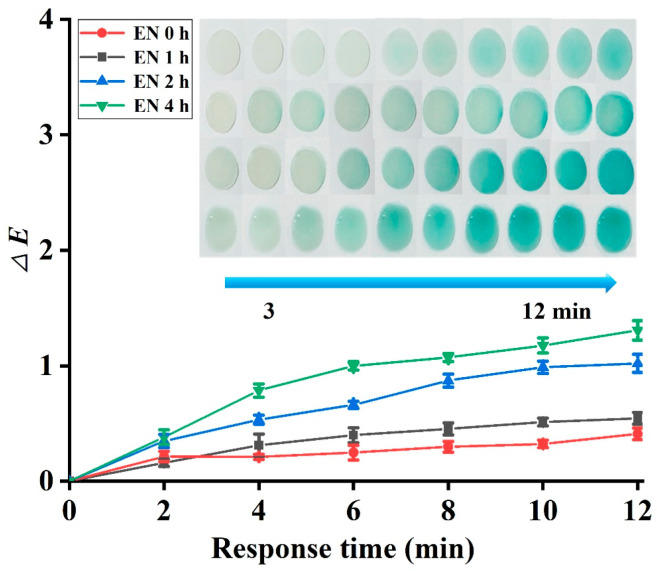
Effect of EN treatment time on the detection performance.

This study aimed to develop a visual detection card for analyzing pesticide residues. The color intensity of the enzyme card after the detection is critical for evaluating the efficacy of the detection card. The decoration time of the fiber mat with EN influences the activation of carboxyl groups in CA, thereby affecting the immobilization of AChE. As illustrated in Figure 4, both enzyme cards prepared with and without EN decoration exhibited a significant color change upon superposition with the SC. It was observed that the color development time decreased with increasing decoration time. In the EN 4 h group, a notable color development was recorded when the EC and the SC were superimposed for 4 min, resulting in the largest change in the ΔE value (1.47). Compared to the undecorated fiber mat, the EN-treated fiber mat primarily facilitates the formation of amide bonds between carboxyls and amines, which enhances enzyme binding and promotes greater color development when superimposed with the SC.

**Figure 5:**
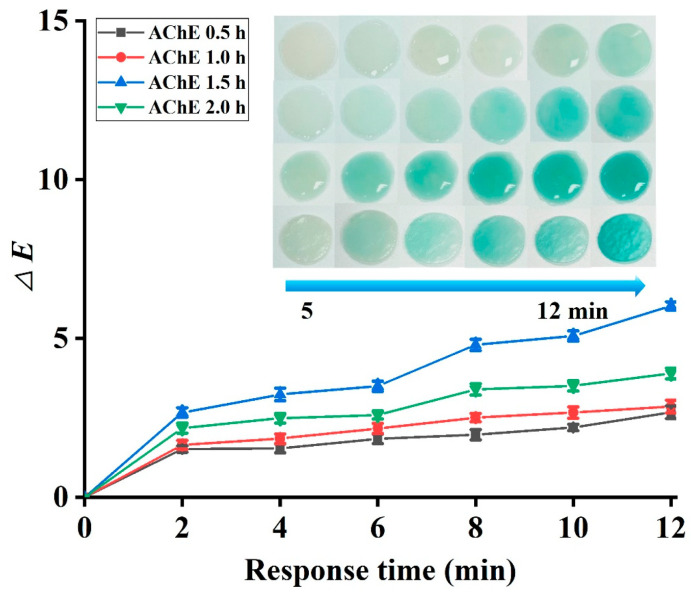
Effect of enzyme immobilization time on the detection performance.

The adsorption time significantly influences the amount of enzyme loaded onto the nanofiber mat, which in turn affects the detection efficacy of pesticides. To investigate this, the activated nanofiber mat was subjected to enzyme adsorption at 4 °C for varying durations, after which its color development was assessed. As illustrated in Figure 5, an increase in adsorption time was associated with a gradual reduction in the color development time of the EC during detection, a phenomenon attributed to the enhanced enzyme adsorption [13]. Furthermore, color difference analysis revealed that the EC prepared through AChE adsorption for 1.5 h displayed a more pronounced color, thereby facilitating better discrimination during pesticide detection. However, extending the adsorption time to 2 h resulted in a less favorable color change of the fiber mat compared to the 1.5 h treatment group. This decline may be attributed to prolonged soaking, which could lead to further swelling of the fiber structure, compromising the integrity of the surface topography and the enzyme loading capacity of the nanofiber mat.

### 3.3. Characterization of Different Nanofiber Mats

#### 3.3.1. FTIR Analysis

**Figure 6:**
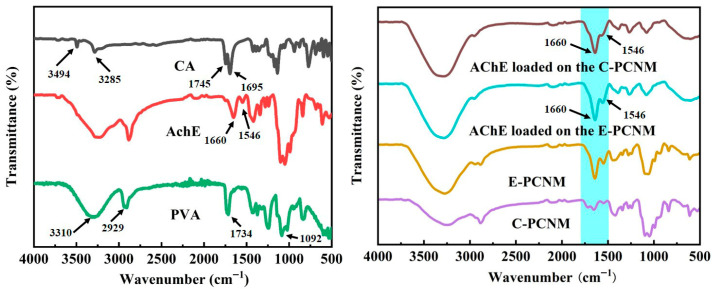
FTIR spectra of CA, AChE, PVA, and different fiber mats.

In this study, ATR-FTIR was employed to analyze potential structural and compositional changes in the fiber mat [27]. As illustrated in Figure 6, characteristic peaks at 3494 and 3285 cm^−1^ in CA correspond to O–H stretching. Additionally, CA displayed strong peaks at 1745 cm^−1^ (C=O stretching) and 1695 cm^−1^ (C–O–H stretching) [28]. The PVA spectrum revealed characteristic peaks at approximately 3310 cm^−1^, 2929 cm^−1^, and 1092 cm^−1^, corresponding to the vibrations of O–H, C–H, and C–O, respectively [29]. The peak at 1734 cm^−1^ was attributed to C–O stretching from residual acetyl groups in PVA [30]. For PCNM, a shift in the wave number for the O–H stretching vibration absorption peak was observed, confirming the hydrogen bond formation and good compatibility between PVA and CA. Furthermore, a reduced peak intensity associated with hydroxyl groups in the spectrum of crosslinked PCNM implied the participation of hydroxyl groups during the crosslinking process [31]. These results collectively demonstrated the successful crosslinking between PVA and CA. Subsequently, the residual carboxyl groups of crosslinked PCNM are activated with EN for the covalent immobilization of AChE. For AChE, the characteristic peaks at 1660 cm^−1^ and 1546 cm^−1^ were associated with the C=O stretching vibration of the amide I band and the N-H stretching vibration of the amide II band, respectively [32]. These bands appeared in both AChE-loaded C-PCNM and the AChE-loaded E-PCNM, indicating the successful immobilization of enzymes on the fiber mat. This phenomenon aligned with findings from other AChE immobilization carriers [33,34]. Moreover, compared to the spectrum of the AChE-loaded C-PCNM, an increase in the intensity of the specific peaks between 1546 cm^−1^ and 1660 cm^−1^ was noted in the spectrum of the AChE-loaded E-PCNM, which may result from a higher amount of AChE immobilized on the fiber mat [35].

#### 3.3.2. Thermal Properties

**Figure 7:**
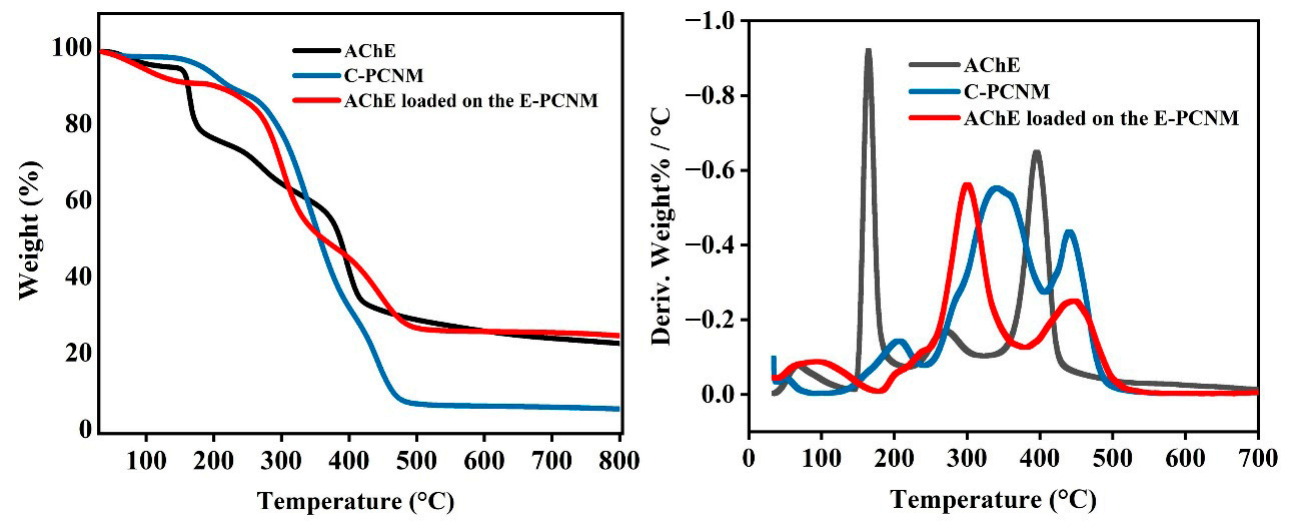
TGA and DTG curves of AChE, C-PCNM, and AChE-loaded E-PCNM.

Thermal stability is a critical property of fiber mats, significantly influencing their structural integrity and performance under high-temperature conditions. This study evaluated the thermal stability and decomposition behavior of nanofiber mats and individual compounds by analyzing their thermogravimetry and derivative thermogravimetry (DTG) curves. As illustrated in Figure 7, the TGA curves of the C-PCNM exhibited four distinct stages of thermal decomposition. In the first stage, thermal degradation occurred within the temperature range of approximately 25 °C to 100 °C, resulting in a weight loss of 2%, which corresponded to the loss of water during heating. The second stage of degradation, occurring between 150 °C and 250 °C, was due to CA decomposition [36]. The third degradation process, spanning from 250 °C to 410 °C, was associated with the degradation of the side chains of PVA, whereas the fourth stage (410 °C to 500 °C) was due to the decomposition of the main backbone chain of PVA [37]. The TGA curve for AChE exhibited three distinct stages of mass loss. The mass loss at 50–200 °C arises from the loss of physically absorbed and bound water in the samples [38]. This observation was in line with previous studies, which indicated that the mass loss occurring from room temperature to 180 °C corresponds to the release of both physically adsorbed and bound water. The peaks observed in the ranges of 200–300 °C and 350–450 °C denoted the degradation of AChE, consistent with earlier findings [12]. Additionally, mass loss occurring at approximately 425 °C has been linked to the degradation of immobilized AChE in another study [39]. In comparison to the degradation peaks of the C-PCNM, the mass loss of the AChE-loaded E-PCNM occurred at a lower temperature, suggesting the degradation of the loaded enzymes, thereby further demonstrating the effectiveness of enzyme immobilization on the fiber membrane [40].

#### 3.3.3. Mechanical Properties

**Figure 8:**
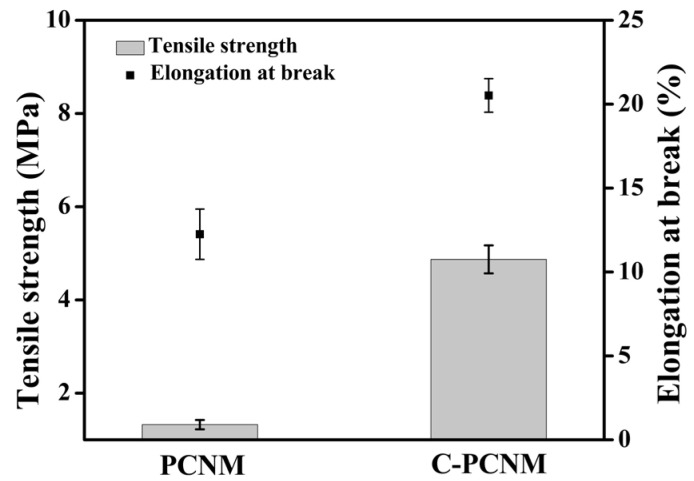
Mechanical properties of nanofiber mats before and after thermal crosslinking (PCNM and C-PCNM).

The mechanical properties of the fiber mat are crucial for maintaining its integrity and withstanding external stress during processing, transportation, storage, and sales [41]. Consequently, the fiber mat must possess sufficient mechanical strength for practical application in pesticide detection cards. The tensile strength and elongation at break are two critical indices for evaluating these mechanical properties. Tensile strength indicates the capacity to resist stretching, while elongation at break measures the ability to stretch before breaking [42]. As illustrated in Figure 8, both the tensile strength and elongation at break of the untreated fiber mat were lower than those of the fiber mat subjected to thermal crosslinking. Specifically, after thermal treatment, the tensile strength and elongation at break of the fiber mat increased by 2.21 times and 1.17 times, respectively. This phenomenon is in agreement with Shi’s study, which reported that tensile strength and elongation at break are functions of CA concentration [43]. Both properties increased following thermal crosslinking at certain concentrations of CA, attributed to the crosslinking process between the functional groups of PVA and CA. An esterification reaction occurs when the PCNM is treated at elevated temperatures, resulting in crosslinks between PVA and CA. These crosslinks are responsible for the enhanced strength of the crosslinked films [44]. Overall, the tensile strength and elongation at break of the thermally crosslinked PCNM were improved, demonstrating its potential applicability for producing detection matrices.

### 3.4. Optimization of the Detection System

**Figure 9:**
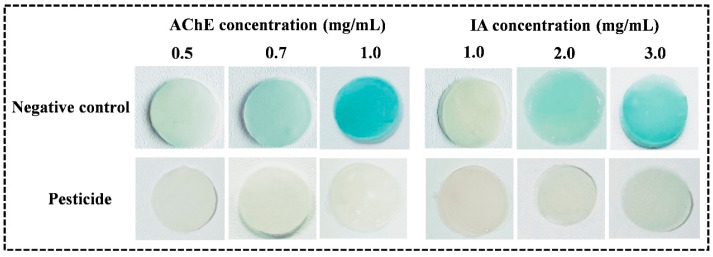
Performance of detection cards fabricated with different concentrations of enzyme and substrate.

In light of economic considerations, minimizing the adsorption of the enzyme in the EC and the adsorption of the substrate agent (IA) on the qualitative filter paper is more advantageous. In this context, the effects of the AChE and IA concentrations on detection efficacy were investigated, with the results illustrated in Figure 9. It was clearly observed that the combination of the EC and SC exhibited a distinct blue color at an enzyme concentration of 1.0 mg/mL. At a concentration of 0.7 mg/mL, the color of the EC remained normal, albeit with reduced intensity. When the enzyme concentration was further decreased to 0.5 mg/mL, the adsorption of the enzyme on the membrane diminished, leading to a light blue color that may be less discernible for the visual detection of pesticide residues. Therefore, an enzyme concentration of 1.0 mg/mL was preferred for the preparation of the EC. Similarly, when the concentration of IA adsorbed by the qualitative filter paper reached 3.0 mg/mL, the color rendering of the detection card was most pronounced. Thus, the optimal selections for the lowest enzyme concentration and substrate concentration were determined to be 1.0 mg/mL and 3.0 mg/mL, respectively.

As illustrated in Scheme 1, the detection time comprises both the inhibition time and the color development time. The IA substrate is catalyzed by AChE, resulting in its degradation into indolephenol, which is subsequently oxidized to form indigo. Pesticides present in the sample can inhibit AChE activity, thereby impeding the development of the blue color. Given that time influences the rate of inhibition, the duration of exposure between the test sample solution and the EC is critical for color development. In this study, samples were applied to the EC for durations ranging from 0 to 12 min, followed by a reaction with the SC. This methodology enabled the assessment of color development results across various inhibition times, optimizing the reaction time between the enzyme and the sample. As shown in Table S3, the positive sample appeared colorless, while the control sample exhibited a distinct blue color when the color development time was set to 8 min and the inhibition time was no less than 4 min. Conversely, if the inhibition time was less than 4 min, enzyme activity persisted due to insufficient inhibition duration, potentially leading to an inaccurate assessment of the positive sample.

In addition to the enzyme inhibition time, the interaction between the EC and the SC also requires a specific duration. The control sample may appear colorless if the color development time is too short, leading to inaccurate results. Conversely, an excessively prolonged color development time may diminish detection efficiency. Therefore, after allowing the sample to react with the EC for 4 min, the subsequent contact with the SC was optimized for a duration ranging from 3 to 15 min. The results presented in Table S4 indicate that the ideal positive sample appeared colorless when the color development time was between 7 and 15 min, while the control sample exhibited a distinct blue color. However, when the color development time was less than 7 min, the results were suboptimal, as the substrate catalyzed by the enzyme was insufficiently oxidized to produce a blue color. Thus, to enhance the detection efficiency, 4 min and 7 min were selected as the appropriate inhibition time and color development time, respectively. The detection performance of other similar tools is depicted in Table 1.

### 3.5. Sensitivity of the Detection Card

To evaluate the efficacy of the self-made card for pesticide detection, the detection limits for phoxim and methomyl were determined. The results of this new card were compared with the standards specified by the Standardization Administration of China [47]. According to national standards, the detection limits for phoxim and methomyl are 0.01 mg/L and 0.15 mg/L, respectively. As presented in Table 2, the rapid detection card was capable of detecting pesticide samples at the national standard detection limits for both pesticides. This indicated that the detection of organophosphorus and carbamate pesticides using the rapid detection card complies with national standards and demonstrates a performance that exceeds these standards to some extent. The detection limits for phoxim and methomyl were found to be 0.007 mg/L and 0.10 mg/L, respectively, which are lower than those reported in a previous study [18]. These findings may be attributed to the superior properties of the designed nanomaterial used as the enzyme-immobilized material.

### 3.6. Storage Stability

Enzyme immobilization is a promising method for preserving bioactivity during storage. Herein, the stability of the as-prepared test cards warrants investigation. Previous research reported that AChE retained 66% of its activity at room temperature after 60 days, suggesting that these cards have a stable storage life of at least two months at room temperature [45]. In this study, a batch of these test cards was prepared and stored at both 4 °C and room temperature to evaluate their storage stability. PBS and two types of pesticides (phoxim and methomyl) were employed as control and positive samples to assess the sensitivity at various intervals, as illustrated in Table 3. The results demonstrated that after 90 days of storage at both 4 °C and room temperature, the rapid test card remained colorless when applied to a positive sample, while the control sample exhibited a blue color. Notably, 70.9% and 66.6% of activity were maintained after 3 months of storage at 4 °C and room temperature, respectively. These findings suggest that the self-made rapid detection card exhibits good storage stability.

### 3.7. Applicability of Detection Card in Food Samples

Organophosphorus and carbamate pesticides are extensively used in the cultivation of fruits and vegetables. In this study, two representative samples, cabbage and orange, were used to evaluate the practical application of this self-made rapid detection card. The results of pesticide detection were compared to those of a commercially available detection card, as presented in Table 4. The test results for cabbage samples treated with 0.2 mg/L methomyl indicated that both the commercial and self-made rapid detection cards remained colorless, demonstrating that both methods could identify methomyl pesticide residues at concentrations within the national standard. However, when the methomyl concentration was reduced to 0.15 mg/L, the self-made card remained colorless while the commercial detection card turned blue. Therefore, the self-made detection card exhibited greater sensitivity than the commercial one for detecting pesticide residues in food. Similarly, the results for the orange samples corroborated those observed in the cabbage samples. Overall, these findings suggested that the practical application of the self-made detection card outperforms the commercial card, particularly in detecting low concentrations of pesticide residues, highlighting its significant potential for ensuring food safety in light of the growing awareness among consumers.

## 4. Conclusions

A fast and sensitive pesticide detection card was developed in this study using an EN-activated thermal crosslinked PCNM as the matrix for the enzyme card. The crosslinked PCNM exhibited unique characteristics and expected water stability, ensuring suitable conditions for the immobilization of AChE. A 2.5-fold increase in enzyme loading capacity on the nanofiber mat was achieved through surface decoration with EDC/NHS (EN) while maintaining 95.7% of its bioactivity after immobilization. Various parameters, including the heating temperature and time, enzyme and substrate concentrations, and inhibition and color development times, were optimized to establish the optimal detection conditions. The developed card demonstrated superior sensitivity and reduced detection time compared to a commercial pesticide detection card. Furthermore, the self-made detection card exhibited good storage stability, with experiments on real food samples verifying its practicality and superiority in pesticide detection compared to the commercial card. These findings highlight the potential of the nanofiber mat for the development of a pesticide detection platform and affirm that the self-made card could serve as a reliable platform for the fast and sensitive detection of food pesticide residues.

## Figures and Tables

**Table 1:** Performance comparisons of similar detection tools.

Card Material	Response Time (min)	Storage Stability (d)	Detection Limits	References
Polyester fiber, glass fiber, and absorbent paper	15	60	0.05, 0.1, 0.5, 0.5, 0.04, and 0.09 mg/mL for phoxim, acephate, malathion, omethoate, carbofuran, and aldicarb	[45]
PVDF	25	60	1.0, 0.1, 2.0, 0.05, 1.5, and 0.8 mg/mL for omethoate, dichlorvos, methamidophos, chlorpyrifos, carbaryl, and pirimicarb	[18]
PVA	15	120	0.5, 1.5, 0.1, and 0.02 mg/mL for omethoate, malathion, carbaryl, and carbofuran	[13]
PLA	20	60	0.2, 1.0, 2.5, and 0.1 mg/mL for Trichlorfon, malathion, carbaryl, and carbofuran	[14]
Filter paper	30	2	0.5, 0.005, 0.1, 0.5, and 0.5 ppm for carbaryl, carbofuran, dichlovos, paraoxon, and pirimicarb	[46]

**Table 2:** Detection limits of two pesticides.

Pesticide	Detection Limit of Rapid Determination (mg/L)	Detection Limit of Rapid Detection Card (mg/L)	Result			
Phoxim	0.01	0.007	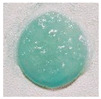	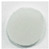	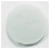	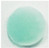
			0	0.01	0.007	0.005
Methomyl	0.15	0.1	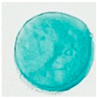	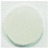	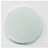	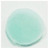
			0	0.15	0.1	0.007

**Table 3:** Storage stability of the rapid detection card.

Sample	Temperature	Storage Time (Days)			
		0	30	60	90
Control	4 °C	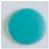	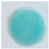	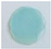	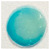
	RT	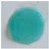	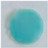	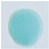	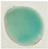
Phoxim	4 °C	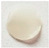	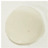	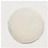	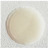
	RT	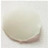	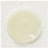	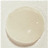	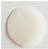
Methomyl	4 °C	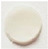	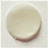	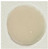	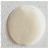
	RT	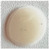	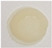	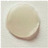	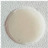

**Table 4:** Comparative evaluation of commercial and self-made cards in food pesticide detection.

Sample	Detection Card	Methomyl Concentration (mg/L)			
		0	0.1	0.15	0.2
Chinese cabbage	Commercial	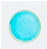	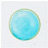	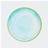	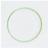
	Self-made	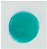	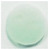	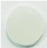	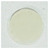
Orange	Commercial	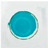	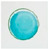	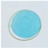	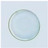
	Self-made	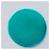	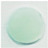	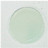	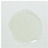

## Data Availability

The original contributions presented in the study are included in the article/Supplementary Material, further inquiries can be directed to the corresponding author.

## Supplementary Materials

The following supporting information can be downloaded at: https://www.mdpi.com/article/10.3390/foods14152628/s1, Figure S1 Influence of thermal crosslinking on microstructure and water contact angle of different electrospun nanofiber mats; Table S1 Properties of electrospinning solutions with different PVA concentrations; Table S2 Water resistance of electrospun nanofiber mat before and after thermal crosslinking; Table S3 The chromogenic results of samples detected with different reaction times; Table S4 The chromogenic results of samples detected with different color development times.

## Abbreviations

The following abbreviations are used in this manuscript:

AChE	acetylcholinesterase
EC	enzyme card
SC	substrate card
PVA	polyvinyl alcohol
CA	citric acid
PNM	PVA nanofiber mat
PCNM	PVA/CA nanofiber mat
EDC	1-ethyl-(3-dimethylaminopropyl) carbodiimide hydrochloride
NHS	N-hydroxysuccinimide
EN	EDC/NHS
C-PCNM	thermally crosslinked PCNM
E-PCNM	EN-treated C-PCNM
IA	indolyl acetate
CD	circular dichroism
SEM	scanning electron microscopy
ATR-FTIR	attenuated total reflection–Fourier transform infrared
ATR	attenuated total reflection
TGA	thermogravimetric analysis
DTG	derivative thermogravimetry
PBS	phosphate-buffered saline
PLA	polylactic acid
