# Supplementary material for: Development of a Rapid and Sensitive Visual Pesticide Detection Card Using Crosslinked and Surface-Decorated Electrospun Nanofiber Mat
Source: Foods. 2025 Jul 26;14(15):2628. doi: 10.3390/foods14152628 (PMC12346702; doi:10.3390/foods14152628)
Supplement: Supplementary file 1 [file foods-14-02628-s001.zip › foods-3738115-supplementary.pdf]

Development of a rapid and sensitive visual pesticide detection card using crosslinked and surface-decorated electrospun nanofiber mat

Yunshan Wei[1], Huange Zhou[2], Jingxuan Kang[2], Yongmei Wu[2,3], Kun Feng[2,3*]

[1]College of Food Science and Engineering, Henan University of Technology, Zhengzhou 450001, China

[2]College of Food and Bioengineering, Zhengzhou University of Light Industry, Zhengzhou, 450001, China

[3]Key Laboratory of Cold Chain Food Processing and Safety Control (Zhengzhou University of Light Industry), Ministry of Education, Zhengzhou, 450001, China

Abbreviations

AChE, acetylcholinesterase; EC, enzyme cards; SC, substrate cards; PVA, polyvinyl alcohol; CA, citric acid; PNM, PVA nanofiber mat; PCNM, PVA/CA nanofiber mat; EDC, 1-ethyl-(3-dimethylaminopropyl) carbodiimide hydrochloride; NHS, N-hydroxysuccinimide; EN, EDC/NHS; C-PCNM, thermally crosslinked PCNM; E-PCNM, EN-treated C-PCNM; IA, indolyl acetate; CD, circular dichroism; SEM, scanning electron microscopy; ATR-FTIR, Attenuated Total Reflection-Fourier Transform Infrared; ATR, attenuated total reflection; TGA, thermogravimetric analysis; DTG, derivative thermogravimetry; PBS, Phosphate-buffered saline; PLA, polylactic acid

*Corresponding author: Email address:fengkun_89@163.com

Supplementary material

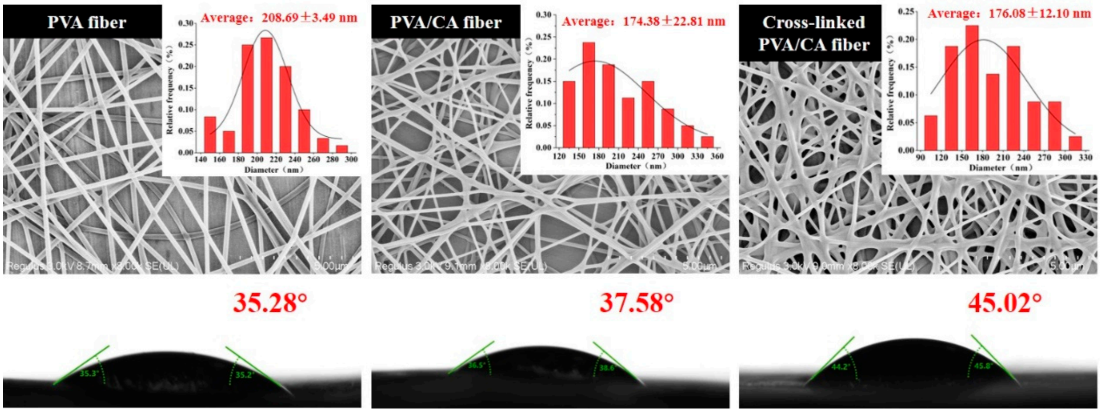

Figure S1 Influence of thermal crosslinking on microstructure and water contact angle of different electrospun nanofiber mats. (electrospinning parameters for PVA fiber: 13 kV, 0.25 mL/h, 11 cm; PVA/CA fiber: 15 kV, 0.4 mL/h, 13 cm)

Table S1 Properties of electrospinning solutions with different PVA concentrations.

Concentration (%, w/v)	Viscosity (cp)	Conductivity (μs/cm)
5.0	ND	254.67±0.58[d]
7.5	184.33±1.53[c]	355.33±2.08[c]
10.0	442.33±2.52[b]	433.00±1.00[b]
12.5	1766.67±6.03[a]	472.67±1.53[a]

Note: The mean ± standard deviation represented by different letters in the same column is significantly different ($P<0.05$), ND means not detected.

Table S2 Water resistance of electrospun nanofiber mat before and after thermal crosslinking.

Treatment group	Water contact Angle (°)	Swelling rate (%)	Absorption loss (%)
PVA	35.28±0.09[b]	ND	100.00±0.00[a]
Before PVA/CA crosslinking	37.58±1.45[b]	ND	100.00±0.00[a]
After PVA/CA crosslinking	45.02±1.12[a]	941.48±73.33	7.59±0.84[b]

Note: The mean ± standard deviation represented by different letters in the same column is significantly different (P<0.05), ND means not detected.

Table S3 The chromogenic results of samples detected with differnt reaction times.

Inhibition time (min)	Positive sample	Control
0	blue	blue
2	blue	blue
4	colorless	blue
6	colorless	blue
8	colorless	blue
10	colorless	blue
12	colorless	blue

Table S4 The chromogenic results of samples detected with different color development times.

Color development time (min)	Positive sample	Control
3	colorless	colorless
5	colorless	colorless
7	colorless	blue
10	colorless	blue
12	colorless	blue
15	colorless	blue
